# Facial expression recognition method based on PSA—YOLO network

**DOI:** 10.3389/fnbot.2022.1057983

**Published:** 2023-01-17

**Authors:** Ruoling Ma, Ruoyuan Zhang

**Affiliations:** ^1^Guangdong Finance and Trade of Vocational College, Guangzhou, China; ^2^Anhui Water Conservancy Technical College, Hefei, China

**Keywords:** YOLOv4 network, PSA—YOLO network, facial expression recognition, channel attention mechanism, operation efficiency

## Abstract

In order to improve the recognition speed and accuracy of face expression recognition, we propose a face expression recognition method based on PSA—YOLO (Pyramids Squeeze Attention—You Only Look Once). Based on CSPDarknet53, the Focus structure and pyramid compression channel attention mechanism are integrated, and the network depth reduction strategy is adopted to build a PSA-CSPDarknet-1 lightweight backbone network with small parameters and high accuracy, which improves the speed of face expression recognition. Secondly, in the neck of the network, a spatial pyramid convolutional pooling module is built, which enhances the spatial information extraction ability of deep feature maps with a very small computational cost, and uses the α—CIoU loss function as the bounding box loss function to improve the recognition accuracy of the network for targets under high IoU threshold and improve the accuracy of face expression recognition. The proposed method is validated on the JAFFE, CK+, and Cohn-Kanade datasets. The experimental results show that the running time of the proposed method and the comparison method is reduced from 1,800 to 200 ms, and the recognition accuracy is increased by 3.11, 2.58, and 3.91%, respectively, so the method proposed in this paper has good applicability.

## 1. Introduction

Nowadays, with the rapid development of computer technology, automatic facial expression recognition technology has been widely applied in networked learning, medical treatment, transportation, and social security fields (Yao et al., 2018; Zhang and He, 2021). Most methods perform expression recognition when the user's head is in the front or near the front state, and the face is basically unaffected by occlusion (Zhang and Xu, 2020). However, this restriction significantly reduces the robustness of the expression recognition algorithm. In addition, there are also some methods to learn user-related facial features by directly constraining users. This feature is particularly sensitive to the identity information of users, so the identification robustness of unknown users' needs to be improved (Lin et al., 2020).

At present, facial expression recognition is mainly divided into two methods: one is a single frame image, and the other is a video image. The former mainly extracts feature images from the input, while the latter can extract the temporal information of the image sequence and the features of each static image (Chen et al., 2018; Tan et al., 2019; Lin et al., 2020). Some facial expression recognition systems may have good performance in some image datasets but poor performance in others, and there is still room to improve the robustness of facial expression recognition (Li et al., 2020; Liu and Xin, 2020). Based on the above analysis, a facial expression recognition method based on PSA—YOLO network is proposed to solve the problems of facial expression recognition accuracy and data set universality.

Facial expressions correspond to a person's internal emotional state, intention, or social information. Literature Jan et al. (2018) defines six basic terms of “anger,” “disgust,” “fear,” “happiness,” “sadness,” and “surprise,” followed by the expression of “contempt.” Facial expression recognition is a traditional problem in computer vision and an essential part of artificial intelligence technology. It has gradually attracted more and more attention, and scholars have proposed a large number of new methods (Islam and Hossain, 2019).

For example, reference Li et al. (2018) proposed a facial expression recognition algorithm combining HOG features and improving KC—FDDL (*K*-means Cluster and Fisher Discrimination Dictionary Learning) Dictionary Learning sparse representation. The HOG features of the normalized expression images were extracted to form the training set, the Fisher discriminant dictionary learning of the improved *K*-means clustering was carried out, and the expression classification was carried out with the sparse representation weighted by the residuals, which overcame the influence of illumination and occlusion in the process of facial expression recognition. However, this method cannot recover sufficient expression information for occluded regions. Literature Tamfous et al. (2020) used sparse coding and dictionary learning methods to study the time-varying shapes in Kendall shape space of 2D and 3D landmarks and studied intrinsic and non-intrinsic solutions to overcome the non-linearity of shape space on facial expression recognition, including action trajectory recognition. However, this method is highly dependent on data sets, and different data sets greatly impact the recognition results (Liu et al., 2020).

In recent years, CNN (Convolutional Neural Networks) has made great contributions to the image classification neighborhood. Many expression recognition methods based on CNN have emerged, which make up for the poor robustness of traditional methods (Wang et al., 2020). For example, a FER (Facial Expression Recognition) method based on a variant feature reduction model and iterative optimization classification strategy was proposed in the literature Du and Hu (2019). WPLBP (Weighted patch-based Local Binary Patterns) is used for feature extraction and expression classification, improving expression recognition accuracy. However, the accuracy of the feature extraction process should be further enhanced. Reference Keyu et al. (2018) proposes a UDADL (Unsupervised Domain Adaptive Dictionary Learning) model, which Bridges the source Domain and target Domain by Learning a shared Dictionary. The analytical dictionary finds approximate solutions as latent variables to simplify the identification process. Literature Liang et al. (2020) proposes a framework for co-learning FER's spatial characteristics and temporal dynamics. The deep network is used to extract spatial features from each frame, the convolution network is used to model the temporal dynamics, and BiLSTM (directional Long Short-Term Memory) network is used to collect clues from the fused functions to complete facial expression recognition. However, the user identity in practice is difficult to define. Literature Chen et al. (2020) proposes a method of facial expression recognition using GAN (Generative Adversarial Network), which focuses on the recognition of facial expressions with a large intra—class gap in the process of facial expression recognition in the real environment so as to better adapt to the tasks with significant intra—class differences.

At present, deep learning-based facial expression target facial expression recognition algorithms are mainly single-stage algorithms with YOLO (You Only Look Once) series as the core and two-stage algorithms with RCNN (Region CNN) as the core (Muhammad et al., 2018). Studies in literature Jin et al. (2019) mainly replace or improve the backbone network in YOLO network to improve the facial expression recognition performance of the algorithm. However, the improved network still has shortcomings, such as insufficient attention to the details of expression images and insufficient utilization of semantic information contained in deep features, affecting the performance of facial expression recognition. Therefore, these factors should be fully considered and utilized to improve the performance of the YOLO network in facial expression and facial expression recognition.

To solve the above problems, Ours takes the YOLOv4 target facial expression recognition network as the basis, aiming at the task of facial expression, facial expression recognition, and aiming at improving the accuracy and speed of facial expression, facial expression recognition by the network, builds PSA—YOLO target facial expression recognition network with the characteristics of high facial expression recognition accuracy, fast facial expression recognition speed, and high facial expression recognition rate of small targets.

## 2. PSA—YOLO recognition algorithm

### 2.1. PSA—YOLO network structure

**Figure 1:**
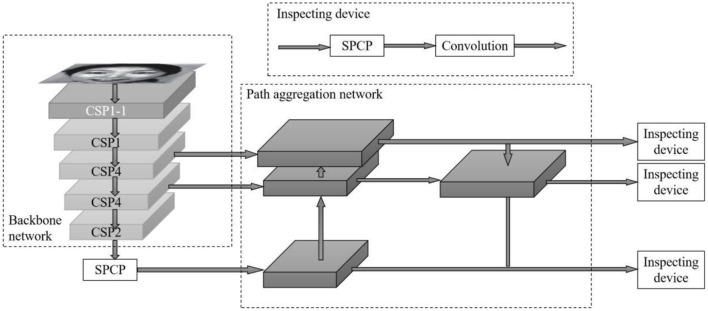
Structure of PSA YOLO.

Ours proposes a PSA—YOLO network based on the YOLOv4 target facial expression recognition network, in which CBM represents “convolution—batch normalization—Mish activation function module” and BN (Batch Normalization), as shown in Figure 1. First, the Focus structure (Glenn, 2021) and PSA mechanism (Zhang et al., 2021) were added to the CSPDarknet53 backbone network, and residual blocks were stacked in the pattern of “1-1-4-4-2” to simplify the number of network layers. Second, SPC (Squeeze and Concat) module and SPP (Spatial Pyramid Pooling) module (He et al., 2014) are fused into SPCSP (Spatial Pyramid Convolution and Pooling) replaces the original SPP module. Finally, the *k*-means clustering method and α-CIOU loss function are used to perform dimension analysis and bounding box regression on the training image, and the facial expression recognition head part remains unchanged. These parts together constitute the basic structure of the PSA—YOLO network.

### 2.2. PSA—CSPDarknet feature extraction network

**Figure 2:**
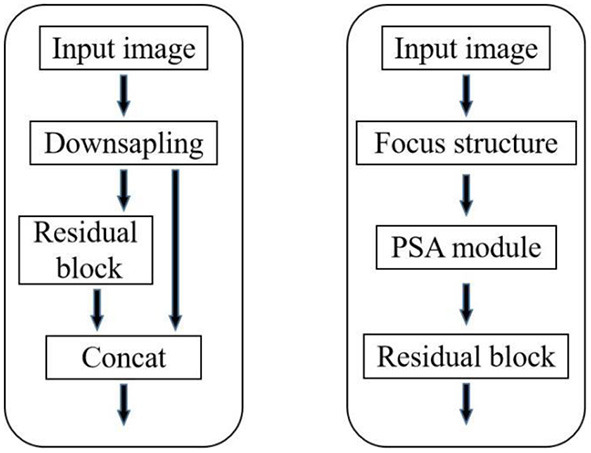
Structure of original CSP 1 1 and improved structure.

To pay more attention to the channels important to target facial expression recognition information in the initial stage of network forward propagation and fully extract the underlying features of facial expression edge texture to improve the accuracy of facial expression recognition, PSA—CSPDarknet network only retains residual blocks in CSP1-1 layer of CSP1-53 network and adds Focus structure and PSA module in front of residual blocks. The PSA—CSPDarkNet network structure is shown in Figure 2. The Focus structure has been used in the YOLOv5 (Hu et al., 2018) target facial expression recognition network to replace the backbone network for the first downsampling, showing good facial expression recognition performance in the COCO dataset. The input image is cut into four similar feature maps by tensor slicing operation, and then the four feature maps are fused in the channel dimension to transform the spatial features into channel features without information loss to replace the first down-sampling in the original network.

**Figure 3:**
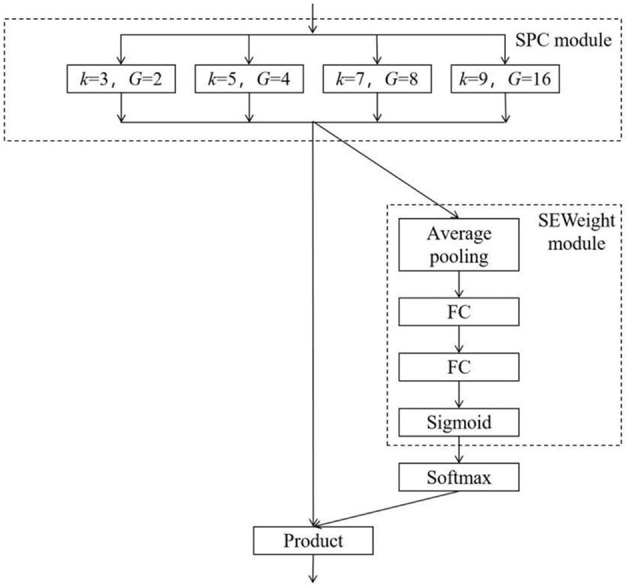
Structure of the PSA mechanism.

The PSA module is divided into four parts, as shown in Figure 3 (*K* represents the convolution kernel size, *G* represents the convolution kernel grouping size, and FC represents the fully connected layer). Firstly, the SPC module effectively extracts and integrates the spatial information of different scales of the input feature map. For the spatial dimension of the input feature map, the SPC module uses convolution kernels of four sizes (3, 5, 7, and 9) to perform grouped convolution. The sizes of the grouped kernels of each size are 2, 4, 8, and 16, respectively, to realize grouped convolution and channel compression of the feature map. Then, the SEWeight module (He et al., 2016) is used to learn the weight of the feature map processed by the SPC module, coordinate the local and global attention, and assign different weights according to the importance of the feature channel to the classification task. Softmax normalizes the weight of the included channel. The interaction between attention weight and the channel is realized by multiplying the normalized weight with the feature map processed by the SPC module so that the channel, which is more important for expression facial expression recognition in the feature map, is assigned with higher weight.

In order to balance the speed and accuracy of the backbone network, based on CSPDarknet53 after the fusion of Focus structure and PSA module, the number of residual blocks is readjusted to simplify the number of network layers and reduce the network parameters and computation burden. Three models were constructed, PSA-CSPDarknet-1, PSA-CSPDarknet-2, and PSA-CSPDarknet-3. Among them, PSA-CSPDarknet-1 halved the number of residual blocks in CSP layer of CSPDarknet53 network and set it as “1-1-4-4-2.” Inspired by the network structures of Resnet-18 and Resnet-34 in literature Liu et al. (2018), PSA-CSPDarknet-2 and PSA-CSPDarknet-3 residual arrangements were set as “1-2-2-2-2” and “1-3-4-6-3,” respectively.

### 2.3. SPCP module

**Figure 4:**
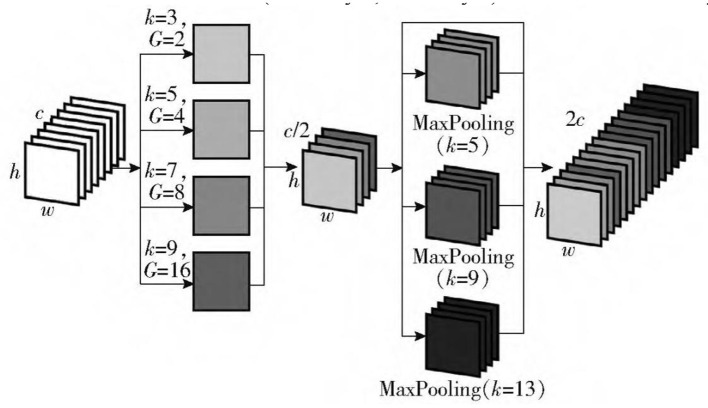
Structure of the SPCP module.

To further extract the multi-scale semantic information of spatial dimensions in the deep backbone network, a spatial pyramid convolution pooling module is constructed to replace the original spatial pyramid pooling module. In YOLOv4, the neck mainly consists of two parts: spatial pyramid pooling and PANet (Path Aggregation Network; Rezatofigh et al., 2019). Spatial pyramid pooling is a particular pooling method, which adopts the maximum pooling with a step size of 1 and convolution kernel size of 5 × 5, 9 × 9, and 13 × 13, which is closely integrated with the feature map of the deepest layer of the backbone network to expand the receptive field and integrate multi-scale spatial information. In PSA—YOLO target recognition network, the backbone network extracts local texture and pattern information to construct the semantic information required by the subsequent layer. However, with the increase in complexity, the width of the network will become larger, especially after the SPP module, the number of convolution kernels reaches 2,048, which increases the number of network parameters and computations. The SPC module is inserted before the SPP module, and the number of convolution kernels entering the SPP module is reduced by half by the method of grouping multi-scale convolution followed by recompression. The network computation is further balanced while the extraction of multi-scale spatial information is strengthened. As shown in Figure 4, before the SPC module is added to the SPP module, the number of channels entering the SPP module is compressed to 1,024 to build the SPCP module. On the premise of not affecting the speed of data propagation in the network, the efficiency of using local feature information and global feature information is improved. The bottom-up path enhancement is used in the path aggregation network to shorten the high-low fusion path of the multi-scale feature pyramid. The feature map information of the CSP4 layer, CSP2 layer, and three scales output by the SPCP module is fused in PSA—CSPDarknet. The feature information of shallow networks (CSP4 layer and CSP2 layer) can be used effectively.

### 2.4. Bounding box loss function

The commonly used bounding box loss functions are evolved based on the IoU loss, such as GIoU (Generalized IoU; Zheng et al., 2020), DIoU (Distance IoU), and CIoU (Complete IoU; He et al., 2021). The α-IoU series loss (Liliana et al., 2019) applies power transformation to summarize the above IOU-based loss. When the noise box with low IoU value appears, the α-IoU loss can adaptively increase the bounding box regression loss value so that the reduction of bounding box loss can be suppressed and the overfitting phenomenon can be avoided when the prediction box with controversy is trained. On the contrary, when the prediction box with high IoU value appears, the α-IoU loss will get lower bounding box loss than the noise box so that the network can predict more objects with high IoU value, and the average accuracy of facial expression recognition at high IoU threshold can be improved. Under the action of the above two factors, the facial expression recognition performance of the network with high IoU threshold will be enhanced.

## 3. Experimental results and analysis

This experiment is based on python1.2 simulation platform, and the hardware environment is: Microsoft Windows 10 operating system, the CPU model is E5-1620 V4, the clock frequency is 3.5 GHz, the graphics card is NVIDIA TITAN V, the video memory size is 12 GB. In this experiment, PSA—YOLO network model was trained for 250 cycles, the minimum batch was 64, and its initial learning rate and learning rate change factor were 0.01 and 0.96, respectively. After each step, the learning rate was reduced. The maximum number of iterations, momentum, and weight decay are 2,000, 0.9, and 0.0002, respectively. After 1,600 iterations, the connections between PSA—YOLO networks have been formed, and the subsequent iterations are trained to enhance correlation and eliminate noise.

### 3.1. JAFFE dataset experiment

**Figure 5:**
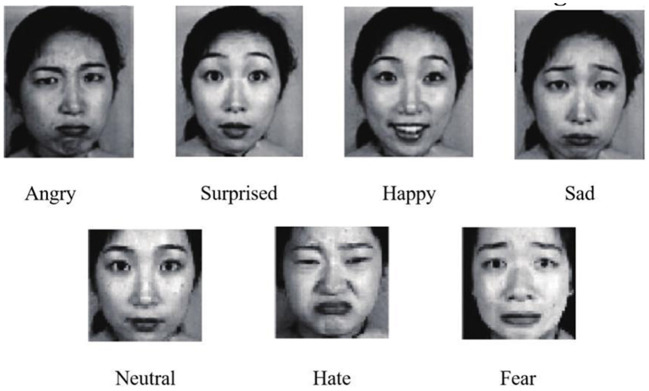
Some image examples of the JAFFE dataset.

JAFFE is a database of facial expressions with just 213 still images. JAFFE dataset is used to test the effect of a small number of images on system training by different training methods. From the JAFFE dataset, 202 images were selected that were processed using image preprocessing techniques (the JAFFE dataset contains some mislabeled facial expressions that were later removed). This dataset has seven different facial expressions: angry, happy, neutral, surprised, sad, afraid and disgusted. A partial image example of the JAFFE dataset is shown in Figure 5.

**Figure 6:**
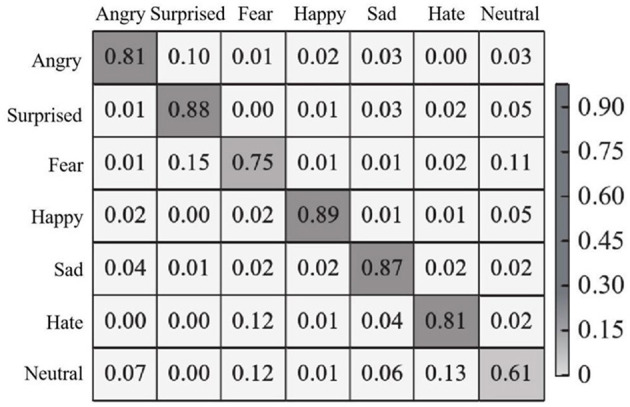
Expression recognition confusion matrixbased on JAFFE dataset.

In each test, 70% of the images were randomly selected as training images, and the remaining images were used as test images. The recognition effect of the proposed method is experimentally demonstrated on the JAFFE dataset. The confusion matrix of seven expressions is shown in Figure 6.

It can be seen from Figure 6 that the recognition accuracy of the proposed method in seven types of facial expressions is all higher than 60%, among which the recognition accuracy of happy, sad and surprised expressions is all higher than 85%, and the happy expression is the easiest to recognize with an accuracy of 89%. Confusion is often caused by the fact that angry and disgusted expressions are similar to each other in some cases, causing them to be indistinguishable in pixel space. In addition, the JAFFE dataset has a small number of images and is suitable for the PSA—YOLO network, so the overall recognition effect is satisfactory. In addition, in the JAFFE data set, the recognition accuracy of each emotion and the overall recognition accuracy obtained by the proposed method and other comparison methods (methods in literature Du and Hu, 2019; Chen et al., 2020; Liang et al., 2020) are shown in Table 1.

**Table 1:** Expression recognition accuracy obtained by different methods in JAFFE dataset.

**Expression**	**Reference Du and Hu (2019)**	**Reference Keyu et al. (2018)**	**Reference Liang et al. (2020)**	**Ours**
Angry	75.02%	77.38%	79.94%	81.02%
Hate	76.39%	78.26%	80.07%	81.95%
Fear	71.88%	73.59%	74.63%	75.78%
Happy	84.39%	86.47%	88.16%	89.01%
Neutral	57.84%	58.91%	60.03%	61.56%
Sad	82.63%	84.19%	86.25%	87.34%
Surprised	83.68%	85.97%	87.35%	88.29%
Average	74.19%	78.56%	80.73%	83.84%

As can be seen from Table 1, both the recognition accuracy of each expression and the overall recognition accuracy, the results obtained by the proposed method are higher than other comparison methods, and the overall recognition accuracy is 83.84%. In literature Du and Hu (2019), WPLBP is used to extract expression features and iterative optimization classification strategy is used to realize expression recognition. However, this method is greatly affected by the extraction accuracy, so the classification accuracy is low, and the overall recognition accuracy is 74.19%. In literature Liang et al. (2020), deep network is used to extract spatial features from each video frame, and facial expression recognition is completed through the BiLSTM network. Face recognition is completed from two perspectives of time and space, with many constraints, and the recognition accuracy is limited to a certain extent. The overall recognition accuracy is 78.56%. In literature Chen et al. (2020), GAN is used to realize facial expression recognition. This method is used primarily to recognize expressions with large intra-class gaps. Therefore, for expressions with small intra-class gaps, the recognition effect is insignificant, such as neutral and fearful expressions.

### 3.2. CK+ dataset experiment

**Figure 7:**
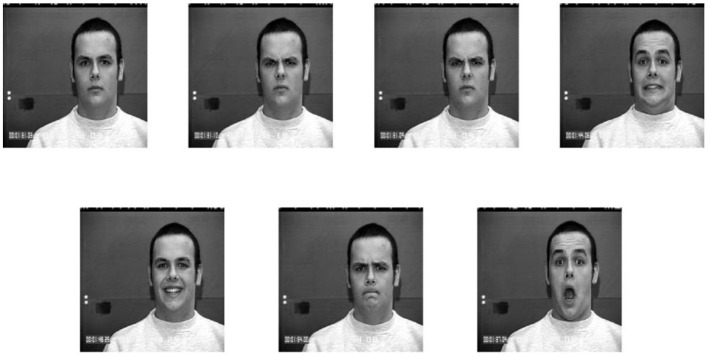
Some image examples of the CK+ dataset.

The CK+ dataset contains 593 facial expression sequences, each of which can be viewed as several consecutive video frames, with ~10,000 facial expression images from 123 models. Since these image sequences are continuous, there are many similar images. In the experiment, 693 images were selected and processed by image preprocessing technology after removing the similar images. Images with seven expressions were selected from the dataset: angry, happy, neutral, surprised, sad, afraid, and disgusted. A partial image example of the CK+ dataset is shown in Figure 7.

**Figure 8:**
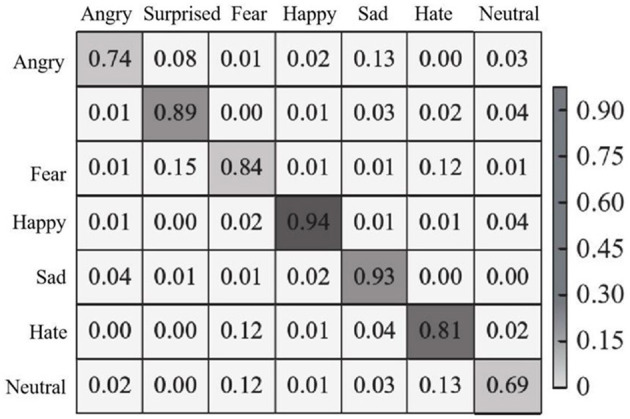
Expression recognition confusion matrix of CK+ dataset.

In each test, 70% of the images were randomly selected as training images, and the remaining images were used as test images. The recognition effect of the proposed method is experimentally demonstrated on the CK+ dataset. The confusion matrix of seven expressions is shown in Figure 8.

As can be seen from Figure 8, the recognition accuracy of the proposed method is higher than 60% in all seven types of facial expressions, among which the recognition accuracy of happy and sad expressions is 94 and 93%, respectively, and the recognition accuracy of surprised, afraid and disgusted expressions is over 80%. Because angry and disgusted expressions are similar to each other in some cases, they are indistinguishable in pixel space, thus confusing. In addition, the large number of images in the CK+ dataset is conducive to model training, so the overall recognition accuracy is high. In addition, in the CK+ data set, the recognition accuracy of each emotion and the overall recognition accuracy obtained by the proposed method and other comparison methods (methods in literature Du and Hu, 2019; Chen et al., 2020; Liang et al., 2020) are shown in Table 2.

**Table 2:** Expression recognition accuracy obtained by different methods in the CK+ dataset.

**Expression**	**Reference Du and Hu (2019)**	**Reference Keyu et al. (2018)**	**Reference Liang et al. (2020)**	**Ours**
Angry	71.24%	72.38%	73.41%	74.17%
Hate	77.45%	78.37%	79.94%	81.23%
Fear	81.86%	82.59%	83.63%	84.58%
Happy	86.91%	88.73%	90.67%	94.49%
Neutral	65.29%	67.67%	68.35%	69.81%
Sad	85.78%	87.56%	89.49%	93.04%
Surprised	85.68%	86.97%	87.35%	89.96%
Average	79.26%	81.14%	82.51%	85.09%

As can be seen from Table 2, the results obtained by the proposed method are higher than other methods in terms of both the recognition accuracy of each emotion and the overall recognition accuracy, with an overall recognition accuracy of 85.09%. Literature Du and Hu (2019) used WPLBP to extract expression features and iteratively optimized the classification strategy to realize expression recognition. Literature Liang et al. (2020) used BiLSTM network combined with deep network to extract spatial and temporal features to complete face recognition. In literature Chen et al. (2020), GAN was used to realize facial expression recognition. Compared with the other three methods, the overall recognition accuracy of the proposed method is improved by 7.32, 4.87, and 3.12%, respectively, which proves the superiority of the facial expression recognition performance.

### 3.3. Cohn-Kanade dataset experiment

**Figure 9:**
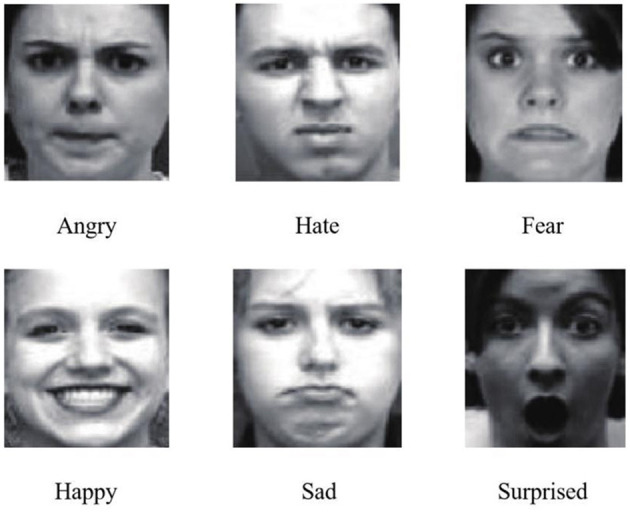
Some image examples of Cohn-Kanade dataset.

The Cohn-Kanade Facial Expression Database was created in 2000 by the Robotics Institute and the Department of Psychology at CMU. The dataset consists of about 500 sequences of multiple expressions from 100 female adults, including African Americans, Latinos, Asians and others. In the experiment, images need to be normalized to obtain images with sizes of 64 × 64. Some images are shown in Figure 9.

**Figure 10:**
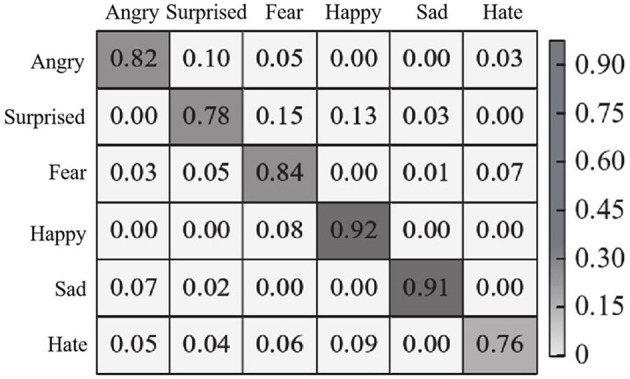
Expression recognition confusion matrix of Cohn-Kanade dataset.

In Cohn-Kanade data set on the experiment, the effect of the method inOurs to identify randomly selected 20 research objects, each object contains six different images of the expression, randomly selected 10 object used in the training, the remaining 10 object is used to test, 30 times to experiment on average, six kinds of expression of the confusion matrix is shown in Figure 10.

As can be seen from Figure 10, the recognition accuracy of each expression of the proposed method is higher than 75%. Since there is no neutral expression in this data set, expressions such as fear and disgust will not be confused with neutral expressions, so the accuracy has been improved to a certain extent. Similarly, happy and sad expressions were easy to recognize, with a recognition accuracy of 92 and 91%, respectively, both higher than 90%. In addition, in the Cohn-Kanade dataset, the recognition accuracy of each emotion and the overall recognition accuracy obtained by the proposed method and other comparison methods (methods in references Du and Hu, 2019; Chen et al., 2020; Liang et al., 2020) are shown in Table 3.

**Table 3:** Expression recognition accuracy obtained by different methods in Cohn-Kanade dataset.

**Expression**	**Reference Du and Hu (2019)**	**Reference Keyu et al. (2018)**	**Reference Liang et al. (2020)**	**Ours**
Angry	78.35%	79.81%	80.41%	82.02%
Hate	72.91%	74.62%	75.49%	76.13%
Fear	81.66%	82.57%	83.36%	84.47%
Happy	87.16%	89.98%	91.74%	92.61%
Sad	86.78%	87.65%	89.49%	91.08%
Surprised	75.02%	76.72%	77.35%	78.25%
Average	78.85%	80.04%	81.68%	84.87%

As can be seen from Table 3, consistent with the recognition structure of JAFFE and CK+ datasets, the proposed method has higher recognition accuracy than other comparison methods in each expression and overall recognition, with an overall recognition accuracy of 84.87%. The recognition accuracy of literature Du and Hu (2019) is greatly affected by the feature extraction accuracy of WPLBP method, so the classification accuracy is not high, and the overall recognition accuracy is 78.85%. Literature Liang et al. (2020) combines the spatiotemporal features of facial expressions and uses convolutional network to model the temporal dynamics, which makes it difficult to extract features. In reference Chen et al. (2020), GAN is used to realize facial expression recognition for expressions with large intra-class gap in the process of facial expression recognition. The application scenario is relatively single, and the recognition effect needs to be improved.

### 3.4. Identify error rates

In order to demonstrate the facial expression recognition performance of the proposed method in the JAFFE data set, CK+ data set and Cohn-Kanade data set, it is compared with the methods in literatures Du and Hu (2019), Chen et al. (2020), and Liang et al. (2020), and the error rate of 5-fold cross-validation is shown in Table 4.

**Table 4:** Error rates in different datasets and different methods.

**Algorithm data set**	**Reference Du and Hu (2019)**	**Reference Keyu et al. (2018)**	**Reference Liang et al. (2020)**	**Ours**
JAFFE (error rate)	26.72%	18.86%	11.08%	8.91%
CK+ (error rate)	21.19%	16.83%	10.95%	5.37%
Cohn-Kanade (error rate)	23.08%	18.15%	10.37%	6.92%

As can be seen from Table 4, the proposed method has the lowest error rate, which is 8.91%. Because the number of images in JAFFE database is very small, deep PSA—YOLO has not yet shown the best performance, so the performance of PSA—YOLO network is close to the recognition effect of GAN used in literature Chen et al. (2020). However, the proposed method adopts PSA—YOLO network model and spatial pyramid convolution pooling module to enhance the spatial information extraction ability of deep feature maps with minimal computational cost, so the expression recognition effect is better. The CK+ dataset, two images were selected for each expression for each subject, one of which was the frame at the beginning of the expression of the emotion, while the other was the frame in the image sequence when the emotion reached its expression peak. The combined classification of the two images can reduce the error rate, so the error rate of the proposed method is reduced compared with the JAFFE dataset. As can be seen from Table 4, the proposed method achieves the lowest error rate of 6.92%. Due to the limited number of images and limited network learning, the error rate of this dataset is higher than that of CK+ dataset, but lower than that of JAFFE dataset due to the lack of neutral expression, which avoids expression confusion.

### 3.5. Other factors affecting the average recognition rate

**Figure 11:**
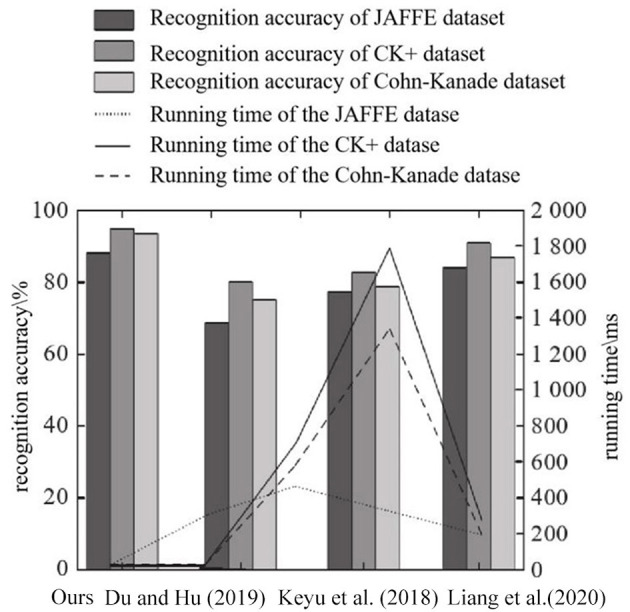
Comparison of running time and recognition accuracy.

In order to further evaluate the performance of the proposed method, it is compared with the methods in literatures Du and Hu (2019), Chen et al. (2020), and Liang et al. (2020) in terms of the running time of the training network and the accuracy of facial expression recognition. The recognition accuracy and running time of different methods on the JAFFE, CK+, and Cohn-Kanade datasets are shown in Figure 11.

**Figure 12:**
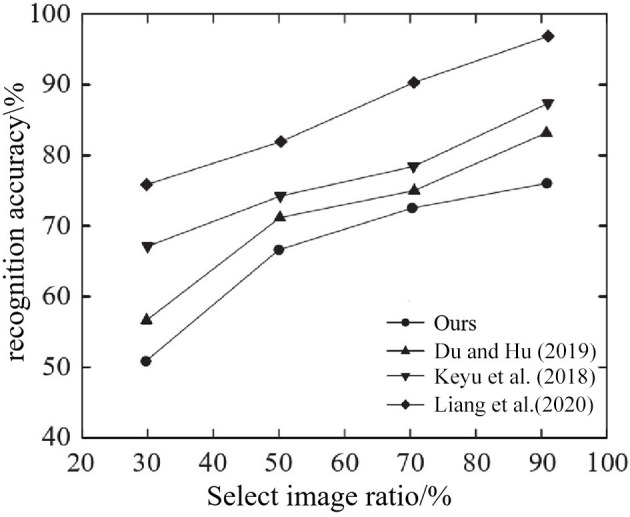
Influence of training image proportion on recognition accuracy of different methods.

As can be seen from Figure 11, on JAFFE, CK+, and Cohn-Kanade datasets, compared with other methods, ours integrates Focus structure and PSA mechanism on the basis of CSPDarknet53, and adopts network depth reduction strategy. A lightweight PSA CSPDarknet 1 backbone network with a small number of parameters and high accuracy was constructed. Secondly, in the neck of the network, a spatial pyramid convolution pooling module is built to enhance the spatial information extraction ability of the deep feature map with minimal computational cost, and the α-CIO U loss function is used as the bounding box loss function to obtain high recognition accuracy. In literature Liang et al. (2020), BiLSTM network combined with spatial and temporal features extracted from deep network is used to complete face recognition and recognize the amount of system data. Therefore, the running time is the longest, which is close to 1,800 ms on CK+ dataset. The WPLBP method in reference Du and Hu (2019) and the GAN model system in reference Chen et al. (2020) are simple in composition, so the running time is reduced compared with that in reference Liang et al. (2020), but the recognition accuracy is lower than that of the proposed method. In addition, the ratio of training images to the images used in the test evaluation enables to evaluate the impact of the ratio of training images of different methods on the selected dataset. In the experiment, 70% of the images in the data set are used as the training set, and the rest are used as the test set. Taking JAFFE database as an example, different proportions of training images using different methods and the resulting recognition accuracies are shown in Figure 12.

As can be seen from Figure 12, when the ratio of training images increases, the recognition accuracy of all methods will improve, and the proposed method shows the best performance regardless of the ratio of training images tested. In fact, when 90% of the images were randomly selected from the JAFFE database as training images and the remaining images were used as the test dataset, the recognition accuracy of the method reached 96.0%.

### 3.6. Ablation experiments

In order to clarify the influence of each network component on classification performance and operational efficiency, an ablation experiment was conducted using the CK+ dataset as an example to test the average accuracy of six expressions of happiness, sadness, anger, surprise, fear and disgust. The proposed method includes four main parts: feature extraction module, PAS module, CSPDdrknet53 module and classification module. Since the classification module is necessary for classification in the network in this paper, the classification module is retained, and the feature extraction module, PAS module, and CSPDdrknet53 module are deleted respectively, and then different experiments are performed, and the results are shown in Table 5. It can be seen that when one of the modules is deleted, the average recognition rate decreases to a certain extent compared to the complete network. Especially in the absence of the feature extraction module, the recognition rate decreased the most, only 51.72%. Generally, the initial features obtained are too coarse, and direct entry into subsequent processing will seriously affect the subsequent results. Therefore, the feature extraction module is required in the network. The PSA module is the core module of the proposed method, and the lack of this module also leads to a serious decrease in the recognition rate, which proves the importance of the PSA module. It can also be seen from Table 5 that without CSPDdrknet53 module, the average recognition rate is 75.12%. Therefore, each module has a certain boost in the final output.

**Table 5:** Ablation experimental results in CK+ dataset.

**Ablation experiment entries**	**Average recognition rate**
None feature extraction module	51.72%
None PSA module	59.07%
None CSPDdrknet53 module	75.12%
Complete network	83.84%

## 4. Conclusion

In order to improve the recognition speed and accuracy of face expression recognition, ours propose a face expression recognition method based on PSA-YOLO. Based on the YOLOv4 network, comparative experiments were carried out on the backbone network, neck, and bounding box loss function. Based on CSPDarknet53, the Focus structure and pyramid compression attention mechanism are added, and the lightweight processing is carried out to build the PSA CSPDarknet backbone network. Secondly, the spatial pyramid convolution pooling module is used in the neck, and the α-CIoU loss is optimized as the bounding box loss function of the expression recognition network. Eventually, the PSA—YOLO network was built. Ablation validation of the proposed method was performed on the JAFFE, CK+, and Cohn-Kanade datasets. The experimental results show that the running time of the proposed method and the comparison method is reduced from 1,800 to 200 ms, and the recognition accuracy is increased by 3.11, 2.58, and 3.91%, respectively, which has obvious recognition advantages.

## Data availability statement

Publicly available datasets were analyzed in this study. This data can be found at: https://tianchi.aliyun.com/dataset?spm=5176.27124976.J_3941670930.19.71de132aItNJg9.

## Author contributions

Both authors listed have made a substantial, direct, and intellectual contribution to the work and approved it for publication.

## References

[B1] Chen J. Xu R. Liu L. (2018). Deep peak-neutral difference feature for facial expression recognition. Multimed. Tools Appl. 77, 29871–29887. 10.1007/s11042-018-5909-533807088

[B2] Chen L. Wu P. Liu Y. T. (2020). Depth learning recognition method for intra-class gap expression. J. Image Graph. 25, 679–687.

[B3] Du L. Hu H. (2019). Weighted patch-based manifold regularization dictionary pair learning model for facial expression recognition using iterative optimization classification strategy. Comput. Vis. Image Understand. 18, 13–24. 10.1016/j.cviu.2019.06.003

[B4] Glenn J. (2021). Yolov5. Available online at: https:github.com/ultralytics/yolov5 (accessed November 22, 2022).

[B5] He J. Erfani S. Ma X. Bailey J. Chi Y. Hua X. S. (2021). “Alpha Io U: A family of power intersection over union losses for bounding box regression,” in Conference and Workshop on Neural Information Processing Systems, 13675.

[B6] He K. Zhang X. Ren S. Sun J. (2014). Spatial pyramid pooling in deep convolutional networks for visual recognition. IEEE Trans. Pat. Anal. Machine Intell. 37, 1904–1916. 10.1109/TPAMI.2015.2389824 26353135

[B7] He K. Zhang X. Y. Ren S. Q. Sun J. (2016). “Deep residual learning for image recognition,” in Conference on Computer Vision and Pattern Recognition (Las Vegas, NV), 770–778. 10.1109/CVPR.2016.90

[B8] Hu J. Shen L. Sun G. (2018). “Squeeze-and-excitation networks,” in Proceedings of the IEEE Conference on Computer Vision and Pattern Recognition, 7132–7141. 10.1109/CVPR.2018.00745

[B9] Islam B. Hossain A. (2019). Fusion of features and extreme learning machine for facial expression recognition. J. Comput. ENCES 15, 1833–1841. 10.3844/jcssp.2019.1833.184133108301

[B10] Jan A. Ding H. Meng H. Chen L. Li H. (2018). “Accurate facial parts localization and deep learning for 3D facial expression recognition,” in Proceedings of the 13th IEEE International Conference on Automatic Face and Gesture Recognition. (Xi'an), 466–472. 10.1109/FG.2018.00075

[B11] Jin X. Wu L. Li X. Zhang X. Chi J. Peng S.. (2019). ILGNet: Inception modules with connected local and global features for efficient image aesthetic quality classification using domain adaptation. IET Comput. Vis. 13, 206–212. 10.1049/iet-cvi.2018.5249

[B12] Keyu Y. Wengming Z. Zhen C. Yuan Z. Tong Z. Chuangao T. (2018). Unsupervised facial expression recognition using domain adaptation based dictionary learning approach. Neuro Comput. 319, 84–91. 10.1016/j.neucom.2018.07.003

[B13] Li M. Peng X. J. Wang Y. (2018). Facial expression recognition based on improved dictionary learning and sparse representation. J. Syst. Simulat. 30, 28–35. 10.16182/j.issn1004731x.joss.201801004

[B14] Li T. T. Hu Y. L. Wei F. L. (2020). Improved facial expression recognition algorithm based on GAN and application. J. Jilin Univ. 58, 163–168. 10.13413/j.cnki.jdxblxb.2019374

[B15] Liang D. Liang H. Yu H. Zhang Y. (2020). Deep convolutional BiLSTM fusion network for facial expression recognition. Vis. Comput. 36, 499–508. 10.1007/s00371-019-01636-3

[B16] Liliana D. Y. Basaruddin T. Widyanto M. R. Oriza I. I. D. (2019). Fuzzy Emotion: A natural approach to automatic facial expression recognition from psychological perspective using fuzzy system. Cogn. Process. 20, 391–403. 10.1007/s10339-019-00923-0 31209637

[B17] Lin K. Z. Bai J. X. Li H. T. Li A. (2020). Facial expression recognition with small samples fused with different models under deep learning. J. Front. Comput. Sci. Technol. 14, 127–137. 10.3778/j.issn.1673-9418.1904028

[B18] Liu F. Li M. Hu J. Xiao Y. Qi Z. (2020). Expression recognition based on low pixel face images. Laser Optoelectron. Progr. 57, 97–104. 10.3788/LOP57.101008

[B19] Liu Q. M. Xin Y. Y. (2020). Face expression recognition based on end-to-end low-quality face images. J. Chin. Comput. Syst. 41, 668–672.

[B20] Liu S. Qi L. Qin H. Shi J. Jia J. (2018). “Path aggregation network for instance segmentation,” in Conference on Computer Vision and Pattern Recognition (Salt Lake City, UT), 8759–8768. 10.1109/CVPR.2018.00913

[B21] Muhammad N. A. Nasir A. A. Ibrahim Z. Sabri N. (2018). Evaluation of CNN, alexnet and GoogleNet for fruit recognition. Indonesian J. Electr. Eng. Comput. Sci. 12, 468–475. 10.11591/ijeecs.v12.i2.pp468-475

[B22] Rezatofigh H. Tsoi N. Gwak J. Sadeghian A. Reid I. Savarese S. (2019). “Generalized intersection over union: A metric and a loss for bounding box regression,” in Conference on Computer Vision and Pattern Recognition (Long Beach, CA), 658–666. 10.1109/CVPR.2019.00075

[B23] Tamfous A. B. Drira H. Amor B. B. (2020). Sparse coding of shape trajectories for facial expression and action recognition. IEEE Trans. Pat. Anal. Machine Intell. 42, 2594–2607. 10.1109/TPAMI.2019.2932979 31395537

[B24] Tan L. Z. Ding Y. Xia L. M. (2019). Facial expression recognition combined with orthogonal neighborhood preserving projection and CNN. J. Chin. Comput. Syst. 40, 2221–2226.

[B25] Wang X. H. Liang Y. C. Ma X. C. (2020). Facial expression classification algorithm research based on ideology of inception. Opt. Technique 46, 94–100.

[B26] Yao Y. Huang D. Yang X. Wang Y. Chen L. (2018). Texture and geometry are scattering representation-based facial expression recognition in 2D+3D videos. ACM Trans. Multimed. Comput. Commun. Appl. 14, 1–23. 10.1145/3131345

[B27] Zhang A. M. Xu Y. (2020). Attention hierarchical bilinear pooling residual network for expression recognition. Comput. Eng. Appl. 56, 161–166.

[B28] Zhang H. Zu K. Lu J. Zou Y. Meng D. (2021). EPSANet: An efficient pyramid squeeze attention block on convolutional neural network. Ar Xiv E-prints. 10.48550/arXiv.2105.14447

[B29] Zhang R. He N. (2021). A survey of micro-expression recognition methods. Comput. Eng. Appl. 57, 38–47.

[B30] Zheng Z. Wang P. Ren D. Liu W. Ye R. Hu Q.. (2020). “Distance Io U loss: Faster and better learning for bounding box regression,” in Conference on Association for the Advancement of Artificial Intelligence (New York, NY), 12993–13000. 10.1609/aaai.v34i07.6999

