# Corrigendum: Facial expression recognition method based on PSA—YOLO network

**DOI:** 10.3389/fnbot.2023.1161411

**Published:** 2023-04-06

**Authors:** Ruoling Ma, Ruoyuan Zhang

**Affiliations:** ^1^Guangdong Finance and Trade of Vocational College, Guangzhou, China; ^2^Anhui Water Conservancy Technical College, Hefei, China

**Keywords:** YOLOv4 network, PSA—YOLO network, facial expression recognition, channel attention mechanism, operation efficiency

In the published article, there was an error in the author list, which was incorrectly ordered. The correctly ordered author list appears below.

“Ruoling Ma^1^ and Ruoyuan Zhang^2*^”

In the published article, there was an error in affiliation 1. Instead of “Guangdong College of Finance and Trade, Guangzhou, China,” it should be “Guangdong Finance and Trade of Vocational College, Guangzhou, China.”

The authors apologize for this error and state that this does not change the scientific conclusions of the article in any way. The original article has been updated.

